# Obesity is associated with senescence of mesenchymal stromal cells derived from bone marrow, subcutaneous and visceral fat of young mice

**DOI:** 10.18632/aging.103606

**Published:** 2020-07-07

**Authors:** Nicola Alessio, Mustafa B. Acar, Ibrahim H. Demirsoy, Tiziana Squillaro, Dario Siniscalco, Giovanni Di Bernardo, Gianfranco Peluso, Servet Özcan, Umberto Galderisi

**Affiliations:** 1 Department of Experimental Medicine, Biotechnology and Molecular Biology Section, Luigi Vanvitelli Campania University, Naples, Italy; 2 Genome and Stem Cell Center (GENKOK), Erciyes University, Kayseri, Turkey; 3 Department of Biology, Faculty of Sciences, Erciyes University, Kayseri, Turkey; 4 Sbarro Institute for Cancer Research and Molecular Medicine, Center for Biotechnology, Temple University, Philadelphia, PA 19122, USA; 5 Research Institute on Ecosystems (IRET), CNR, Italy

**Keywords:** obesity, mesenchymal stromal cells, cell cycle, senescence, differentiation

## Abstract

White adipose tissue (WAT) is distributed in several depots with distinct metabolic and inflammatory functions. In our body there are subcutaneous (sWAT), visceral (vWAT) and bone marrow (bWAT) fat depots. Obesity affects the size, function and inflammatory state of WATs. In particular, obesity may affect the activity of mesenchymal stromal cells (MSCs) present in WAT. MSCs are a heterogeneous population containing stromal cells, progenitor cells, fibroblasts and stem cells that are able to differentiate among adipocytes, chondrocytes, osteocytes and other mesodermal derivatives.

In the first study of this kind, we performed a comparison of the effects of obesity on MSCs obtained from sWAT, vWAT and bWAT. Our study showed that obesity affects mainly the biological functions of MSCs obtained from bone marrow and vWAT by decreasing the proliferation rate, reducing the percentage of cells in S phase and triggering senescence. The onset of senescence was confirmed by expression of genes belonging to RB and P53 pathways.

Our study revealed that the negative consequences of obesity on body physiology may also be related to impairment in the functions of the stromal compartment present in the several adipose tissues. This finding provides new insights as to the targets that should be considered for an effective treatment of obesity-related diseases.

## INTRODUCTION

Senescence, which arrests cell division and produces loss of cellular functions, is induced by several stressors, such as telomere shortening, DNA damage, elevated mitogenic signals, alteration of chromatin organization, metabolic perturbations. Senescence can arrest cancer growth by blocking the proliferation of transformed cells, but it can also contribute to organismal aging [1, 2]. Recently, there has been evidences showing that senescence is a major mechanism in the development and progression of various diseases and this may include metabolic diseases such as obesity, type-2 diabetes, liver steatosis, ischemic injury [3–6].

Obesity is a complex disease that causes white adipose tissue (WAT) dysfunction and is associated with cardiovascular, chronic inflammatory and other metabolic pathologies [7]. In physiological conditions, WAT has a key role in body energy homeostasis by storing fatty acids and releasing them when fuel is required. It contributes to the regulation of glucose metabolism and exerts endocrine functions by secreting dozens of adipocyte-derived factors (adipokines), which are involved in inflammation, regulation of food intake, energy expenditure, reproductive function and pro- and anti-apoptotic activity [7, 8]. WAT is distributed in several depots that have distinct metabolic and inflammatory functions. In our body there are subcutaneous (sWAT) and visceral (vWAT) depots. Obesity also determines alterations in bone marrow microarchitecture by increasing the proportion of bone marrow fat (bWAT), which plays a key role within the bone microenvironment, mainly as a source of adipokines that regulate hematopoiesis and bone homeostasis [9].

Obesity affects the size, function and inflammatory state of WATs and, as a consequence, alters the stem cell niches present in these tissues. These changes induce remodeling of the stem cells present in the niches. In particular, mesenchymal stromal cells (MSCs) could modify their biological properties. This phenomenon may have profound consequences for health given the role of MSCs in bone, cartilage and fat tissue regeneration and in the body’s homeostasis.

MSCs are a heterogeneous population containing stromal cells, progenitor cells, fibroblasts and stem cells. MSCs have been isolated from the stromal component of several tissues and organs, such as bone marrow, adipose tissue, cord blood and dental pulp [10]. Stem cells present in MSC population are able to differentiate among adipocytes, chondrocytes, osteocytes and other mesodermal derivatives. MSCs contribute to the homeostatic maintenance of many organs through secretion of many cytokines and growth factors, which exert paracrine and autocrine functions [10].

In this scenario, we aimed to evaluate how obesity may affect the *in vitro* biological functions of MSCs derived by sWAT, vWAT and bone marrow. We focused our attention on senescence phenomena associated with obesity status. Some studies have addressed the effect of obesity on senescence. There is a recent finding showing that bone marrow derived MSCs from obese individuals are prone to senescence [11]. Other studies, evidenced that adipose tissue may undergo senescence phenomena [4]. Nevertheless, a comparative study on senescence phenomena in MSCs obtained from different sources is still lacking [12–15].

## RESULTS

### In obese mice, the visceral fat- and bone marrow-derived MSCs showed a reduction in proliferation rate and an increase in senescence

**Figure 1:**
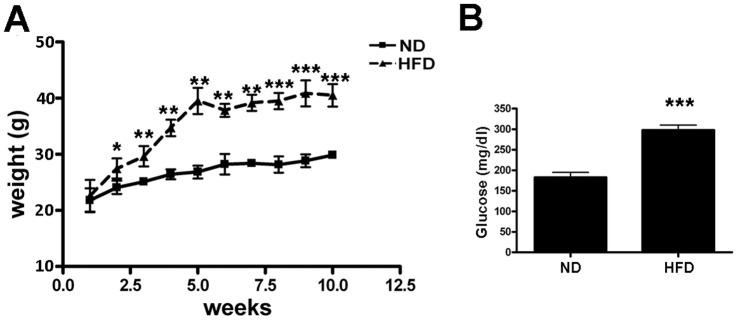
**Treatment of mice with HFD.** (**A**) The graph shows the weight of six mice fed with HFD and other six ones with ND for 10 weeks. Data are shown with standard deviation (SD) n=6 animals for each experimental condition, *p<0.05, **p<0.01, ***p<0.001. (**B**) The graph shows the mean blood glucose levels determined in mice at the end of treatment with either HFD or ND. Data are shown with standard deviation (SD) n=6 animals for each experimental condition, ***p<0.001.

The high-fat diet (HFD) induced a significant increase in mice weight (Figure 1A), with the presence of abundant vWAT depots. As reported, HFD treatment caused hyperglycemia as determined by blood glucose measurement (Figure 1B). We isolated and cultivated MSCs from BM, sWAT and vWAT of obese and normal mice and determined their *in vitro* properties. We verified by flow cytometry that MSCs expressed the surface antigens CD73, CD90 and CD105 and were negative for CD45, CD31 (Supplementary Figure 1) [16].

**Figure 2:**
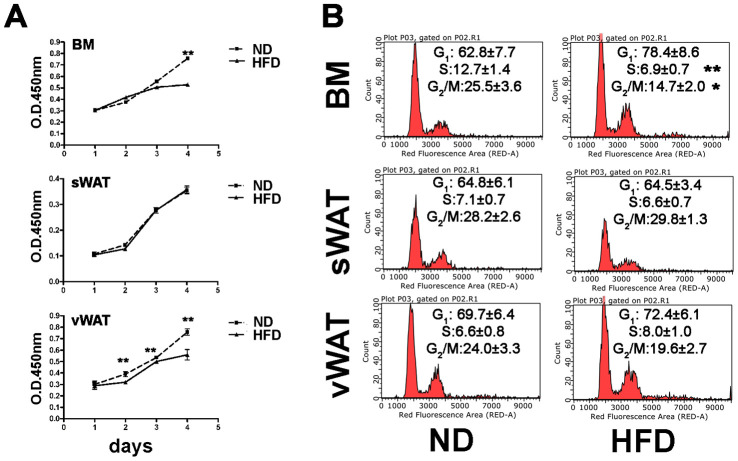
**Proliferation and cell cycle analyses.** (**A**) MSC cell proliferation was evaluated by Cell Counting Kit-8 (CCK-8) colorimetric assay. The graph shows data coming from obese and control samples. Data are shown with standard deviation (SD) n=6 animals for each experimental condition, **p<0.01. (**B**) Representative cell cycle analysis of MSCs harvested from obese and normal mice. Data are expressed with SD (n=6 animals for each experimental condition) *p<0.05, **p<0.01.

In samples obtained from obese animals, the proliferation assay showed a reduction in the proliferation rate of BM- and vWAT-MSCs, while those obtained from sWAT did not show changes compared with controls (Figure 2A). This data agreed with cell cycle profile analysis only for BM-MSCs, which revealed a reduction of S-phase cells (Figure 2B). In the vWAT-MSC samples, obtained from obese animals, we hypothesized that the G_0_/G_1_ fraction could contain more cells in G_0_ rather than in G_1_, this to explain the reduction in proliferation. This assumption is supported by senescence data (see below).

**Figure 3:**
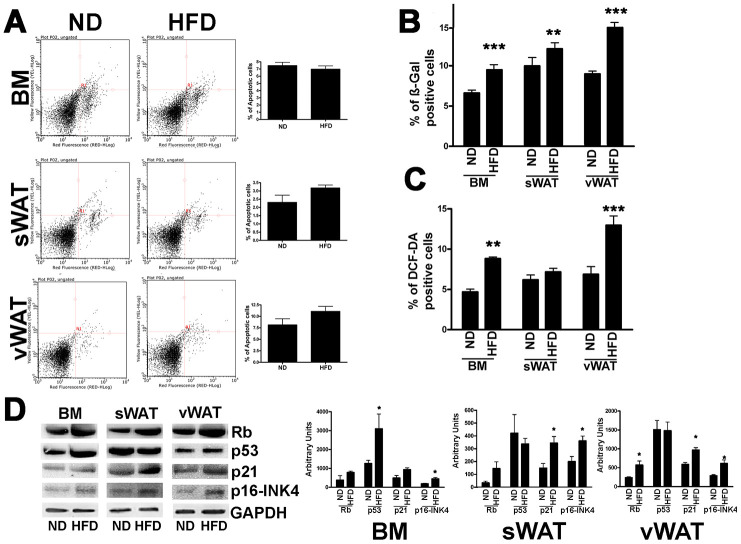
**Apoptosis and senescence in MSCs from obese and control animals.** (**A**) Representative FACS analysis of MSC apoptosis. The assay identifies early (Annexin V + and 7ADD −) and late apoptosis (Annexin V + and 7ADD +). Apoptosis is a continuous process and we calculated the percentage of apoptosis as the sum of early and late apoptotic cells. The histogram shows the mean percentage of Annexin V-positive cells. Data are expressed with standard deviation (n=6 animals for each experimental condition). (**B**) The graph shows mean percentage value of senescent cells determined by SPiDER-ßgal assay. Data are expressed with SD (n=6 animals for each experimental condition) **p<0.01, ***p<0.001. (**C**) The graph shows mean percentage value of cells showing discrete amount of intracellular ROS that are above the threshold detected by H2DCFDA assay. Data are expressed with SD (n=6 animals for each experimental condition) **p<0.01, ***p<0.001. (**D**) Western blot analysis of proteins regulating senescence. The picture shows a representative blot analysis the expression levels of Rb, p53, p21, p16/INK4, Gapdh (loading control). The graph shows mean expression levels (±SD, n = 6 biological replicates, *p<0.05).

We then evaluated apoptosis and senescence by annexin V and acid-beta-galactosidase assays, respectively (Figure 3A, 3B). MSCs obtained from obese animals did not evidence a change in apoptosis levels compared with controls. However, the percentage of senescent cells was greater in MSCs from obese mice compared with those from normal animals (Figure 3B). In particular, the senescence level in vWAT-MSCs was significantly higher in obese samples than in normal controls. An increase in the production and release of reactive oxygen species (ROS) is a typical feature of senescent cells [17, 18]. In MSC cultures obtained from obese animals, we detected a significant increase in intracellular ROS levels (Figure 3C).

### RB and P53 pathways are activated in MSCs obtained from obese mice but not in controls

The decrease in cell proliferation along with the trigger of senescence were consistent with an increase in the levels of RB1, P21, and P16, which play key roles in regulation of the cell cycle and senescence (Figure 3D) [19]. In detail, in obese BM-MSCs, P16 showed the highest expression change (p<0.05); in sWAT-MSCs both P16 and P21 showed significant expression difference (p<0.05); while in obese vWAT-MSCs, the RB and P16 expression was statistically upregulated (p<0.05).

Of note, in vWAT- and sWAT-MSCs from obese mice, the cell cycle exit and senescence were not related to P53, as we did not observe an increase in its expression. On the contrary, the P53 protein level was significantly upregulated in BM-MSCs obtained from obese animals (Figure 3D).

### DNA damage repair following stress

**Figure 4:**
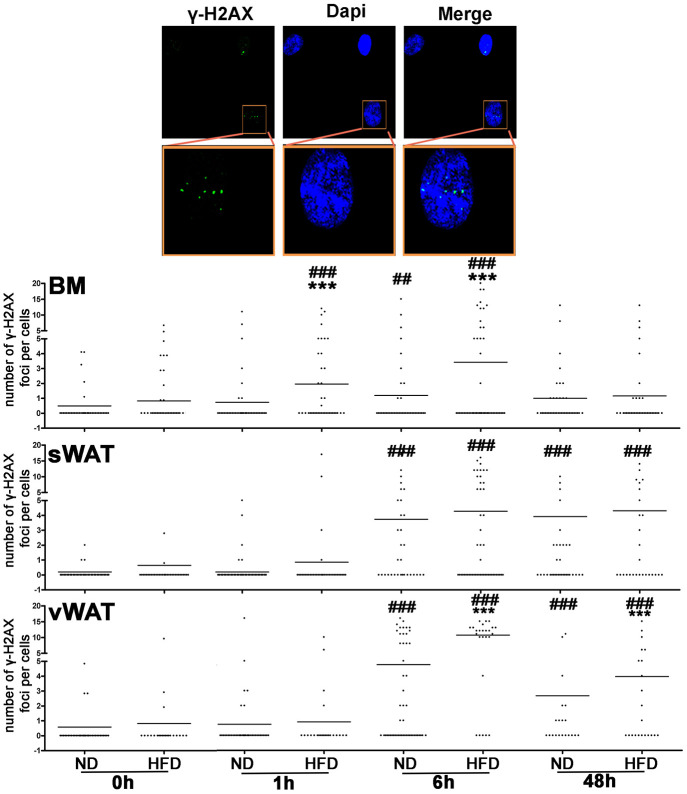
**DNA damage following genotoxic stress in MSCs from obese and control animals.** The pictures are representative images of immunocytochemistry performed on MSC samples to detect γ-H2AX (green) and nuclei (blue). Insets, are higher magnifications of the same samples. The column scatter plot indicates the degree of H2AX phosphorylation that was determined by counting the number of γ-H2AX immunofluorescent foci per cell. The foci number was determined for 200 cells. Each dot represents a single cell. Horizontal bars indicate the mean value for each category (n = 6 biological replicates; ***p <0.001 is statistical significance between ND and HFD samples; ^###^ p<0.001 is statistical significance within a single group, either ND or HFD. The comparison is between time zero and the other time points).

The presence of senescence phenomena in MSCs may suggest that the DNA repair system cannot cope with all the genotoxic injuries occurring during a lifetime. We determined the effectiveness of DNA repair machinery by inducing DNA damage with hydrogen peroxide (H_2_O_2_) treatment and evaluated the expression level of histone γ-H2AX in order to assess the repair capacity of MSCs following genotoxic stress (Figure 4). DNA damage induces the activation of ATM that promotes H2AX phosphorylation. The phosphorylated isoform (γ-H2AX) contributes to recruit and/or retain DNA repair proteins on DNA damage foci. The γ-H2AX foci in nuclei are signs of damaged DNA that are being repaired. Soon after DNA damage events, the presence of these foci is a sign of active repair, while foci persistence several hours or days after stress reveal the presence of unrepaired or misrepaired DNA [20, 21].

We performed a follow-up of γ-H2AX foci 1 hr, 6 hrs and 48 hrs following 300 μM H_2_O_2_ treatment of MSC cultures. BM-MSCs from control mice showed a strong increase of γ-H2AX staining 6 hours after stress and then a decline of damage foci. In samples from obese animals, the peak of DNA damage foci was detected 1 hour after stress, and then the signal intensity decreased. Of interest in sWAT- and vWAT-MSCs from both control and obese mice, we noticed a persistence of γ-H2AX foci even at 6 hrs and 48 hrs time point, suggesting the presence of unrepaired/misrepaired DNA.

### Stemness and differentiation properties

**Figure 5:**
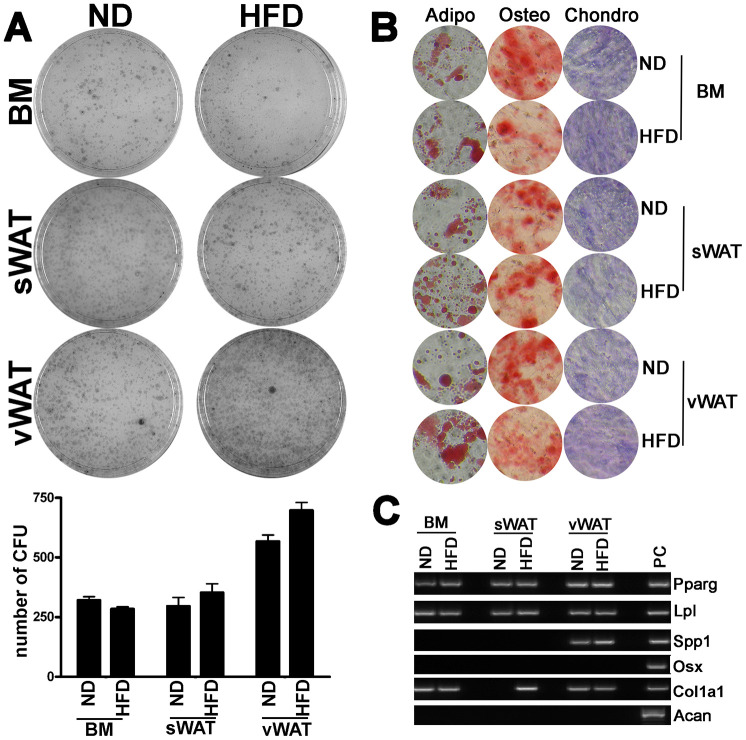
**Clonogenic and differentiation properties of MSCs.** (**A**) The pictures show representative crystal violet staining (CFU-assay) of clones obtained after 14 days of incubation, with MSCs obtained from obese and control animals. The mean number of clones per 1,000 cells plated in 100 mm dish (± SD, n = 6) is indicated in the graph below. (**B**) Adipocyte, osteocyte, and chondrocyte differentiation of MSCs obtained from obese and control animals. The figure shows representative images of Oil Red Oil (adipocytes), Alizarin Red S (osteocytes), and Safranin O (chondrocytes) staining for every experimental condition. (**C**) Qualitative RT-PCR analysis of differentiation markers expressed in proliferating MSC cultures from obese and normal samples. The picture shows a representative gel-electrophoresis analysis.

Senescence could affect the MSC properties, such as self-renewal and multipotential differentiation properties. CFU assay, however, did not show significant changes in the clonogenic potential of MSCs from obese samples compared with controls (Figure 5A). Moreover, the MSC differentiation into adipocytes, chondrocytes and osteocytes, following incubation in induction media, was not impaired in obese mice (Figure 5B).

MSCs are a heterogeneous population containing both stem cells and lineage-committed progenitors for production of osteocytes, chondrocytes and adipocytes. Hence, in the absence of external differentiation cues, in proliferating MSC cultures it is possible to detect markers of lineage commitment. The identification of such markers is an indication of which lineage-committed progenitors are more abundant and/or are already at an advanced differentiation stage in the MSC population. In proliferating MSCs from BM, sWAT and vWAT of control animals, we observed the expression of adipogenic (LPL, PPARG) and osteogenic markers (Osteopontin/SPP1, Osterix/Osx), while we did not detect the expression of chondrogenic markers (Aggrecan/Acan, Col1A1) (Figure 5C). The obesity status did not affect the expression of these markers (Figure 5C).

## DISCUSSION

Senescence occurring in tissues and organs may have negative consequences on the body’s functions. This event may greatly impair the renewal of tissues and the organismal homeostasis, especially when it affects stem cells, such as those present in MSCs. Several studies have shown that MSCs reside at the outer surface of the sinusoids in the bone marrow stroma. This finding supports the hypothesis that multipotent stromal cells could be present in the subendothelial region of small blood vessels present in the stromal component of nearly every organ [22]. It is evident that senescence of MSCs may have multi-organ aftermaths.

MSCs can be also isolated from stromal components of adipose tissues and play a key role in adipose tissue development, differentiation and maintenance as well as in organismal homeostasis [10]. Recent studies have evidenced that obesity may induce senescence of bone marrow derived MSCs [11]. These researches are consistent with findings showing that senescence is a major mechanism in the development and progression of various metabolic diseases, such as obesity and type-2 diabetes [3].

On this premise, we performed a more systematic investigation on the impact of obesity on the functionality of MSCs obtained from different districts. We evaluated obesity effects on MSCs obtained from sWAT, vWAT and bWAT of young male mice. Analysis of senescence is not a trivial investigation, since several *in vitro* and *in vivo* parameters may change the research outcomes. For example, *in vitro* cultivation may affect MSC properties [23]. For this reason, we performed our investigation on cells at very early passages (P1-P3). Furthermore, many *in vivo* parameters (sex, age, co-morbidities) may impair a meaningful readout of senescence process. We then selected only young male animals to reduce variability, since chronological aging and estrogen fluctuation may influence MSC functions [24–26]. Further studies should be performed to evaluate the effects of age and sex on MSC dysfunction associated with obesity.

Our study demonstrated that obesity affected mainly the biological functions of MSCs obtained from BM and vWAT with a reduction in proliferation rate, reduced percentage of cells in S phase and a trigger of senescence. The onset of senescence was confirmed by expression of genes belonging to RB and P53 pathways. The MSCs obtained by sWAT appeared less affected by obesity status, even if an increase in senescence phenomena was detected.

The onset of senescence may be related to ROS production in a self-sustaining loop: ROS may induce senescence and then senescent cells produce ROS [27]. Of note, we observed a significant increase of intracellular ROS concentration in MSCs obtained from obese animals compared with controls. The increase in ROS may be one of the ways obesity may affect MSC functions: high levels of circulating lipids and glucose can produce an excess of energy substrates for cellular metabolic pathways, which in turn increase ROS production.

The DNA repair capacity of MSCs is not significantly affected by obesity status in BM-MSCs. On the contrary, we observed a difference in the time course increase of γ-H2AX between control and obese samples. In these latter ones, the γ-H2AX upregulation occurred earlier than in control samples.

Soon after DNA damage events, cells have to modify their chromatin status in order to allow the DNA repair. This phenomenon may suggest that the DNA repair machinery in obese samples is already active on DNA, given the presence of DNA damages at time 0. Indeed, in obese BM-MSCs we detected high ROS levels that are responsible of genotoxic injuries.

In MSCs obtained from sWAT and vWAT of both normal and obese mice, we observed a permanence of unrepaired DNA foci following genotoxic stress, suggesting that MSCs coming from these fat depots are less proficient in coping with DNA damage stress than BM-MSCs. This result should also be further investigated for the therapeutic implications it may have. Indeed, in recent years, sWAT has been used as an alternative source to BM to obtain MSCs for cell therapy treatment. A note of caution should be sounded on this alternative if it will be proved that MSCs obtained from sWAT are less effective in repairing DNA compared with BM-MSCs. This note of caution could be added to the other concerns about the use adipose tissue derived MSCs. Indeed, MSCs from adipose tissues exhibited procoagulant activity, due to the expression of TF/CD142 factor, which mediates the activation of blood coagulation cascade and complement system [28, 29]. This event may have possible lethal effects upon MSC infusion.

Senescence associated with obesity did not change both the number of CFU clones and the presence of adipo- osteo- and chondro-progenitors, as evidenced by RT-PCR on proliferating MSC cultures. This result suggests that senescence may affect the final part of differentiation process rather than cell self-renewal and cell commitment.

Indeed, Wu and co-workers performed a semi-quantitative analysis of osteo-chondro-adipo differentiation of MSCs from obese and normal mice. They found that obesity greatly influence MSC differentiation [15]. On the contrary, it remains to be established if MSC differentiation that is affected in obese mice can produce mature and functional adipocytes, osteocytes and chondrocytes. This investigation requires further *in vivo* studies along with physiological analysis on differentiated cells.

In conclusion, our study has demonstrated that the negative consequences of obesity on body physiology may also be related to impairment in the functions of the stromal compartment present in several adipose tissues. This observation further reinforces the concept that obesity is a disease as designated by the American Medical Association (AMA) in 2013. Alteration of the stromal component may have profound and deleterious consequences on body functions, since stem cells that promote tissue renewal and homeostasis are located in this stromal component.

## MATERIALS AND METHODS

### Animals

C57BL/6 inbred male mice of 3 weeks of age were purchased from Charles River (Wilmington, MA, USA). The study on animals was approved by Italian Ministry of Health (n. 317-2016PR) and animals were handled in compliance with the protocols that were approved by the Animal Care and Use Committee of Luigi Vanvitelli Campania University. After arrival, the mice were divided into two groups and were fed either a high-fat diet (HFD) (Research Diets, New Brunswick, NJ, USA) or a normal diet (ND) for 10 weeks. At the end of this treatment, the animals were sacrificed and tissue samples were harvested for the experiments indicated below.

The high-fat diet consisted of 60% fat from lard, 20% carbohydrate, and 20% protein (total 5.21 kcal/g), whereas the normal diet contained 10% fat, 70% carbohydrate, and 20% protein (total 3.82 kcal/g). Food intake and body weight were measured once a week till the end of the experiments.

### Glucose measurement

At the end of high and normal fat treatments, blood glucose levels were determined in fasted animals by tail bleeding using a Contour blood glucose meter (Ascensia Diabetes Care, Parsippany, NJ, USA) according to the manufacturer’s instructions.

### Mouse MSC isolation and CFU assay

We harvested MSCs from the bone marrow of the femurs and tibias of mice by inserting a 21-gauge needle into the shaft of the bone and flushing it with alpha-MEM. The cells from one animal were plated onto two 100-mm dishes with alpha-MEM containing 15% FBS. After 48 hours, we discarded the nonadherent cells and washed the adherent cells with PBS 1X. We then incubated the cells for 7 to 10 days in a proliferating medium in order to reach confluence (P0). The cells were then trypsinized and were seeded for the acid beta galactosidase assay.

We collected MSCs from 500 mg sWAT surrounding the hips of animals and from 1 g of vWAT obtained from the abdomen area. Tissues were digested in a DMEM solution containing collagenase type II (1mg/ml) for 1 hour at 37 °C. Samples were filtered on cell strainers (70 μm mesh), centrifuged and washed three times with PBS 1X. Cells were plated onto 100-mm dishes with alpha-MEM containing 15% FBS. We then incubated the cells for 7 days in a proliferating medium in order to reach confluence (P0). The cells were then trypsinized and were seeded for the acid beta galactosidase assay.

An aliquot of cells at P1 was used for the CFU assay. In brief, 1,000 cells were plated on 60 mm plates and were incubated for 15 days without a medium change. The plates were collected, fixed and stained with 0.5% crystal violet. The stained colonies were identified under a light microscope and were counted.

### Adipogenic differentiation

MSCs were treated for 15 days in mesenchymal stem cell adipogenic differentiation medium (PT-3004-KT; Lonza, Walkersville, MD, USA). The medium contains insulin (recombinant), dexamethasone, indomethacin and 3-isobuty-l-methyl-xanthine (IBMX). Lipid droplets were revealed by staining with Oil Red O.

### Osteogenic differentiation

MSCs were treated for 15 days in mesenchymal stem cell osteogenic differentiation medium (PT-3002-KT; Lonza). The medium contains dexamethasone, ascorbate and glycerophosphate. Staining with Alizarin Red S revealed calcium deposits in differentiated osteocytes.

### Chondrogenic differentiation

MSCs were seeded as a pellet in 96 round-bottom multi-wells and cultured in a chondrogenic medium composed of DMEM, 1% FBS, 50 nM ascorbate-2-phosphate (Sigma-Aldrich, St. Louis, MO, USA), 0.1 mM dexamethasone (Sigma-Aldrich, MO, USA), and 10 ng/mL human transforming growth factor (hTGF)-β1 (PeproTech, London, UK). After 21 days, Safranin O staining was performed to detect glycosaminoglycan formation on the cell surfaces.

### Cell proliferation assay

Cell proliferation was determined by Cell Counting Kit-8 (CCK-8) colorimetric assay (Dojindo Molecular Technologies, Kumamoto, Japan). We seeded 5,000 cells in 96-wells and CCK-8 reagents were added. The viability was detected by a microplate reader at 450 nm 24 hrs, 48 hrs and 72 hrs after the incubation.

### Cell cycle analysis

For each analysis, 5x10^4^ cells were collected by trypsin treatment and then, after the PBS washes, were fixed in 70% ethanol overnight at -20 °C. The samples were then washed with PBS 1X and finally were dissolved in a hypotonic buffer containing propidium iodide (Sigma-Aldrich, MO, USA). The samples were acquired on a Guava EasyCyte flow cytometer (Merck Millipore, Danvers, MA, USA) and analyzed through a standard procedure using EasyCyte software.

### In situ senescence-associated acid beta galactosidase assay

Cells grown in flasks were fixed using a solution of 2% formaldehyde and 0.2% glutaraldehyde for 5 min at RT. After that, the cells were washed with PBS 1X (MicroGem, Naples, Italy) and then incubated with SPiDER-ßgal kit a (Dojindo Molecular Technologies). The samples were acquired on a Guava EasyCyte flow cytometer (Merck Millipore) and analyzed through a standard procedure using EasyCyte software.

### Apoptosis detection

Apoptosis was detected using a fluorescein-conjugated Annexin V kit (Dojindo Molecular Technologies) on a Guava EasyCyte flow cytometer (Merck Millipore) following the manufacturer’s instructions.

### Treatment with DNA-damaging agent and immunocytochemistry for γ-H2AX detection

MSC cultures were treated for 30 min with 300 μM H_2_O_2_. Following treatment, the medium was removed, and a complete medium was added. Cells were then collected for data analysis 1, 6 and 48 hrs later.

The histone γ-H2AX (2577, Cell Signaling, Danvers, MA, USA) was detected according to manufacturer’s protocol. DAPI staining was performed, and then the cells were observed through a fluorescence microscope (Leica Italia, Italy). The degree of H2AX phosphorylation (γ-H2AX) was evaluated by counting the number of gamma-H2AX immunofluorescent foci per cell. A foci number was determined for 200 cells.

### Reactive oxygen species detection

The intracellular reactive oxygen species (ROS) level was analyzed by using the H2DCFDA test (Thermo Fisher Italia, Monza, Italy) according to the manufacturer’s instructions. The ROS derivatives were quantified on a Guava EasyCyte flow cytometer (Merck Millipore) following the manufacturer’s instructions.

### Western blot (WB) analysis

Cells were lysed in a buffer containing 0.1% Triton (Bio-Rad, Irvine, CA, USA) for 30 min in ice. Then, 20 μg of each lysate was electrophoresed in a polyacrylamide gel and electroblotted onto a nitrocellulose membrane. We used the following primary antibodies: RB1 (AV33212) and GAPDH (G8795) were from Sigma-Aldrich (MO, USA), RB2/P130 (R27020) was from BD Biosciences (San Jose, CA, USA), p27^KIP1^ (3686) was from Cell Signaling, p107 (sc-318), p53 (sc-126), and p21^CIP1^ (sc-397) were from Santa Cruz Biotechnology (Dallas, TX, USA), and p16^INK4A^ (ab54210) was from Abcam (Cambridge, UK). Immunoreactive signals were detected with a horseradish peroxidase-conjugated secondary antibody (ImmunoReagents, Raleigh, NC, USA) and reacted with ECL plus reagent (Merck Millipore). All of the antibodies were used according to the manufacturer’s instructions. The mean value was quantified densitometrically using Quantity One 1-D analysis software (Bio-Rad).

### Qualitative RT-PCR

Total RNA was extracted from cell cultures using Omnizol (EuroClone, Pero, Italy). The mRNA levels were measured by RT-PCR with 5X Al-In-One RT MasterMiX (ABM, Richmond, BC, Canada) as reported previously [30]. PCR cycles were adjusted to include linear amplification for all of the targets. Each RT-PCR reaction was repeated at least three times. A qualitative analysis of mRNA levels was performed with the Gel Doc UV System (Bio-Rad), as already reported [16].

### Statistical analysis

Statistical significance was determined using one-way ANOVA and post-hoc tests by JASP, which is is an open-source statistics software supported by the University of Amsterdam (https://jasp-stats.org). All statistical analysis data are in Supplementary File 1.

## Supplementary Material

Supplementary Figure 1

Supplementary File 1
