# Supplementary material for: Obesity is associated with senescence of mesenchymal stromal cells derived from bone marrow, subcutaneous and visceral fat of young mice
Source: Aging (Albany NY). 2020 Jul 7;12(13):12609–21. doi: 10.18632/aging.103606 (PMC7377882; doi:10.18632/aging.103606)
Supplement: Supplementary Figure 1 [file aging-12-103606-s001..pdf]

SUPPLEMENTARY FIGURE

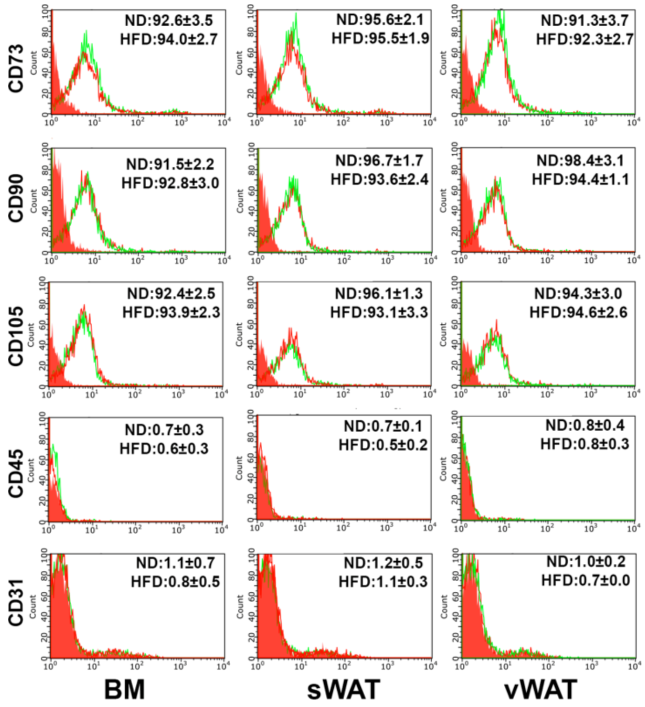

Supplementary Figure 1. Flow cytometry analysis of MSCs. BM-, vWAT- and sWAT-MSCs were trypsinized, washed with PBS and incubated with either anti-CD105 FITC (sc-18893 FITC) or anti-CD90 PE (SC-1914 PE) or anti-CD73 PE (sc-398260) or anti-CD45 Alexa 488 (sc-19597 AF488) or anti-CD31 Alexa 594 (sc-376764 AD594). The antibodies were used according to the manufacturer procedures (Santa Cruz Biotechnology CA, USA). After 30 min of incubation with the antibodies at room temperature, cells were washed with PBS and resuspended in FACS buffer for data acquisition on a Guava EasyCyte flow cytometer (Merck Millipore MA, USA). We performed data analysis with a standard procedure using EasyCyte software. A minimum of 5,000 cells per sample were analysed and gated for forward scatter (FSC) versus side scatter (SSC) channel signals. In each panel, the empty histograms show positive cells from obese (red) and control animals (green) and the filled red histograms represent the negative isotype. ND = Normal Diet treated animals; HFD = High Fat diet treated animals.
