# Supplementary material for: Obesity is associated with senescence of mesenchymal stromal cells derived from bone marrow, subcutaneous and visceral fat of young mice
Source: Aging (Albany NY). 2020 Jul 7;12(13):12609–21. doi: 10.18632/aging.103606 (PMC7377882; doi:10.18632/aging.103606)
Supplement: Supplementary File 1 [file aging-12-103606-s002..pdf]

Supplementary File 2
Statistical analysis

ANOVA Figure 1A

ANOVA

Cases	Sum of Squares	df	Mean Square	F	p
V1	21.66	1	21.66	1.958	0.234
Residual	44.24	4	11.06		

Note. Type III Sum of Squares

Post Hoc Tests

Post Hoc Comparisons

		Mean Difference	SE	t	p_{tukey}	
HFD 1w	ND 1w	3.800	2.715	1.399	0.234	

Post Hoc Comparisons

		Mean Difference	SE	t	p_{tukey}	
HFD 2w	ND 2w	8.867	2.122	4.179	0.014	**

Post Hoc Comparisons

		Mean Difference	SE	t	p_{tukey}	
HFD 3w	ND 3w	9.967	1.368	7.286	0.002	**

Post Hoc Comparisons

		Mean Difference	SE	t	p_{tukey}	
HFD 4w	ND 4w	10.00	1.365	7.326	0.002	**

Post Hoc Comparisons

		Mean Difference	SE	t	p_{tukey}	
HFD 5w	ND 5w	11.50	1.617	7.114	0.002	**

Post Hoc Comparisons

		Mean Difference	SE	t	p_{tukey}	
HFD 6w	ND 6w	13.07	1.619	8.071	0.001	**

Post Hoc Comparisons

		Mean Difference	SE	t	p_{tukey}	
HFD 7w	ND 7w	12.97	1.790	7.244	0.002	**

Post Hoc Comparisons

		Mean Difference	SE	t	p_{tukey}	
HFD 8w	ND 8w	13.93	1.384	10.07	< .001	***

Post Hoc Comparisons

		Mean Difference	SE	t	p_{tukey}	
HFD 9w	ND 9w	12.73	0.784	16.24	< .001	***

Post Hoc Comparisons

		Mean Difference	SE	t	p_{tukey}	
HFD 10w	ND 10w	13.50	1.552	8.696	< .001	***

ANOVA Figure 1B

ANOVA - Glucose

Cases	Sum of Squares	df	Mean Square	F	p
V1	25090.7	1	25090.7	118.5	< .001
Residual	846.7	4	211.7		

Note. Type III Sum of Squares

Post Hoc Tests

Post Hoc Comparisons - V1

		Mean Difference	SE	t	p_{tukey}	
HFD	ND	129.3	11.88	10.89	< .001	***

ANOVA Figure 2A - BM

ANOVA - CCK-8

Cases	Sum of Squares	df	Mean Square	F	p
ND;0	202.8	1	202.8	1.202	0.353
Residual	506.0	3	168.7		

Note. Type III Sum of Squares

Post Hoc Tests

Post Hoc Comparisons - ND

		Mean Difference	SE	t	p_{tukey}
HFD 24h	ND 24h	−13.00	11.86	−1.097	0.353

Post Hoc Comparisons - ND;

		Mean Difference	SE	t	p_{tukey}
HFD 48h	ND;48h	2.333	3.677	0.635	0.571

Post Hoc Comparisons - ND

		Mean Difference	SE	t	p_{tukey}
HFD 72h	ND 72h	−8.333	3.361	−2.479	0.089

Post Hoc Comparisons - ND;0 ▼

		Mean Difference	SE	t	p_{tukey}
HFD 96h	ND 96h	−30.33	4.238	−7.157	0.006 **

ANOVA Figure 2A sWAT

ANOVA - CCK-8

Cases	Sum of Squares	df	Mean Square	F	p
ND;0	13.33	1	13.33	0.375	0.584
Residual	106.67	3	35.56		

Note. Type III Sum of Squares

Post Hoc Tests

Post Hoc Comparisons - ND

		Mean Difference	SE	t	p_{tukey}
HFD 24h	ND 24h	3.333	5.443	0.612	0.584

Post Hoc Comparisons - ND

		Mean Difference	SE	t	p_{tukey}
HFD 48h	ND 48h	−6.167	5.557	−1.110	0.348

Post Hoc Comparisons - ND

		Mean Difference	SE	t	p_{tukey}
HFD 72h	ND 72h	14.67	8.735	1.679	0.192

Post Hoc Comparisons - ND

		Mean Difference	SE	t	p_{tukey}
HFD 96h	ND 96h	0.017	0.026	0.651	0.551

ANOVA Figura 2A vWAT

ANOVA - CCK-8

Cases	Sum of Squares	df	Mean Square	F	p
V1	4.167e −4	1	4.167e −4	0.962	0.382
Residual	0.002	4	4.333e −4		

Note. Type III Sum of Squares

Post Hoc Tests

Post Hoc Comparisons - V1

		Mean Difference	SE	t	p_{tukey}	
HFD **24h**	ND **24h**	−0.017	0.017	−0.981	0.382	

Post Hoc Comparisons - V1

		Mean Difference	SE	t	p_{tukey}	
HFD**48h**	ND **48h**	−0.053	0.011	−5.060	0.007	**

Post Hoc Comparisons - V1

		Mean Difference	SE	t	p_{tukey}	
HFD **72h**	ND **72h**	−0.037	0.012	−2.940	0.042	**

Post Hoc Comparisons - V1

		Mean Difference	SE	t	p_{tukey}	
HFD **96h**	ND **96h**	−0.317	0.040	−7.862	0.001	**

ANOVA Figure 2B - BM

ANOVA – cell cycle

Cases	Sum of Squares	df	Mean Square	F	p
V1	214.8	1	214.80	3.708	0.126
Residual	231.7	4	57.93		

Note. Type III Sum of Squares

Post Hoc Tests

Post Hoc Comparisons –

		Mean Difference	SE	t	p_{tukey}	
HFD **G1**	ND **G1**	11.97	6.215	1.926	0.126	

Post Hoc Comparisons – V1 ▼

		Mean Difference	SE	t	p_{tukey}	
HFD **S**	ND **S**	−5.800	0.904	−6.418	0.003	**

Post Hoc Comparisons – V1

		Mean Difference	SE	t	p_{tukey}	
HFD **G2/M**	ND **G2/M**	−10.80	2.378	−4.542	0.010	*

ANOVA Figure 2B - sWAT

ANOVA – cell cycle

Cases	Sum of Squares	df	Mean Square	F	p
V1	0.144	1	0.144	0.006	0.942
Residual	97.745	4	24.436		

Note. Type III Sum of Squares

Post Hoc Tests

Post Hoc Comparisons

		Mean Difference	SE	t	p_{tukey}
HFD G1	ND G1	−0.310	4.036	−0.077	0.942

Post Hoc Comparisons

		Mean Difference	SE	t	p_{tukey}
HFD S	ND S	−0.500	0.572	−0.875	0.431

Post Hoc Comparisons

		Mean Difference	SE	t	p_{tukey}
HFD G2/M	ND G2/M	1.600	1.678	0.953	0.394

ANOVA Figure 2B - vWAT

ANOVA – cell cycle

Cases	Sum of Squares	df	Mean Square	F	p
V1	10.94	1	10.94	0.280	0.625
Residual	156.34	4	39.09		

Note. Type III Sum of Squares

Post Hoc Tests

Post Hoc Comparisons

		Mean Difference	SE	t	p_{tukey}
HFD G1	ND G1	2.700	5.105	0.529	0.625

Post Hoc Comparisons

		Mean Difference	SE	t	p_{tukey}
HFD S	ND S	−0.500	0.739	−0.676	0.536

Post Hoc Comparisons

		Mean Difference	SE	t	p_{tukey}
HFD G2/M	ND G2/M	−4.400	2.462	−1.787	0.148

ANOVA Figure 3A

ANOVA - Annexin

Cases	Sum of Squares	df	Mean Square	F	p
V1	0.042	1	0.042	0.305	0.610
Residual	0.547	4	0.137		

Note. Type III Sum of Squares

Post Hoc Tests

Post Hoc Comparisons

		Mean Difference	SE	t	p_{tukey}
HFD BM	ND BM	−0.167	0.302	−0.552	0.610

Post Hoc Comparisons

		Mean Difference	SE	t	p_{tukey}
HFD sWAT	ND sWAT	0.667	0.397	1.678	0.169

Post Hoc Comparisons

		Mean Difference	SE	t	p_{tukey}
HFD vWAT	ND vWAT	2.833	1.175	2.411	0.073

ANOVA Figure 3B

ANOVA – Beta Gal

Cases	Sum of Squares	df	Mean Square	F	p
V1	45.375	1	45.375	109.3	< .001
Residual	1.660	4	0.415		

Note. Type III Sum of Squares

Post Hoc Tests

Post Hoc Comparisons

		Mean Difference	SE	t	p_{tukey}	
HFD BM	ND BM	5.500	0.526	10.46	< .001	***

Post Hoc Comparisons

		Mean Difference	SE	t	p_{tukey}	
HFD sWAT	ND sWAT	2.300	0.327	7.042	0.002	**

Post Hoc Comparisons

		Mean Difference	SE	t	p_{tukey}	
HFD vWAT	ND vWAT	5.500	0.526	10.46	< .001	***

ANOVA Figure 3C

ANOVA - DCF-DA

Cases	Sum of Squares	df	Mean Square	F	p
V1	71.415	1	71.415	61.65	0.001
Residual	4.633	4	1.158		

Note. Type III Sum of Squares

Post Hoc Tests

Post Hoc Comparisons

		Mean Difference	SE	t	p_{tukey}	
HFD BM	ND BM	6.900	0.879	7.852	0.001	**

Post Hoc Comparisons

		Mean Difference	SE	t	p_{tukey}
HFD sWAT	ND sWAT	0.800	0.309	2.588	0.061

Post Hoc Comparisons

		Mean Difference	SE	t	p_{tukey}	
HFD vWAT	ND vWAT	4.333	0.464	9.333	< .001	***

ANOVA Figure 3D - BM

ANOVA – WB

Cases	Sum of Squares	df	Mean Square	F	p
V1	252150	1	252150	2.865	0.166
Residual	352065	4	88016		

Note. Type III Sum of Squares

Post Hoc Tests ▼

Post Hoc Comparisons

		Mean Difference	SE	t	p_{tukey}
HFD **Rb**	ND **Rb**	410.0	242.2	1.693	0.166

Post Hoc Comparisons

		Mean Difference	SE	t	p_{tukey}
HFD **p53**	ND **p53**	1078	313.5	3.437	0.041 *

Post Hoc Comparisons

		Mean Difference	SE	t	p_{tukey}
HFD **p21**	ND **p21**	443.0	159.0	2.785	0.050

Post Hoc Comparisons

		Mean Difference	SE	t	p_{tukey}
HFD **p16**	ND **p16**	261.7	65.69	3.983	0.016 *

ANOVAFigure 3D -sWAT

ANOVA - WB

Cases	Sum of Squares	df	Mean Square	F	p
V1	36038	1	36038	2.567	0.184
Residual	56161	4	14040		

Note. Type III Sum of Squares

Post Hoc Tests

Post Hoc Comparisons

		Mean Difference	SE	t	p_{tukey}
HFD **Rb**	ND **Rb**	155.0	96.75	1.602	0.184

Post Hoc Comparisons

		Mean Difference	SE	t	p_{tukey}
HFD **p53**	ND **p53**	−85.33	151.4	−0.564	0.603

Post Hoc Comparisons

		Mean Difference	SE	t	p_{tukey}	
HFD **p21**	ND **p21**	195.3	61.80	3.161	0.034	*

Post Hoc Comparisons

		Mean Difference	SE	t	p_{tukey}	
HFD **p16**	ND **p16**	161.0	54.02	2.980	0.041	*

ANOVA Figure 3D - vWAT

ANOVA - WB

Cases	Sum of Squares	df	Mean Square	F	p
V1	165668	1	165668	9.495	0.037
Residual	69789	4	17447		

Note. Type III Sum of Squares

Post Hoc Tests

Post Hoc Comparisons

		Mean Difference	SE	t	p_{tukey}	
HFD **Rb**	ND **Rb**	332.3	107.8	3.081	0.037	*

Post Hoc Comparisons

		Mean Difference	SE	t	p_{tukey}
HFD **p53**	ND **p53**	−29.33	331.6	−0.088	0.934

Post Hoc Comparisons

		Mean Difference	SE	t	p_{tukey}	
HFD **p21**	ND **p21**	385.3	84.70	4.549	0.010	*

Post Hoc Comparisons

		Mean Difference	SE	t	p_{tukey}	
HFD **p16**	ND **p16**	321.3	97.45	3.297	0.030	*

ANOVA Figure 4 - BM

ANOVA – H2AX

Cases	Sum of Squares	df	Mean Square	F	p
V1	14.680	7	2.097	109.4	< .001
Residual	0.307	16	0.019		

Note. Type III Sum of Squares

Post Hoc Tests

Post Hoc Comparisons

		Mean Difference	SE	t	p_{tukey}
HFD 0h	HFD 1h	−0.800	0.113	−7.077	< .001 ###
	HFD 48h	−0.033	0.113	−0.295	1.000
	HFD 6h	−2.300	0.113	−20.347	< .001 ###
	ND 0h	0.233	0.113	2.064	0.474
	ND 1h	−0.033	0.113	−0.295	1.000
	ND 48h	0.133	0.113	1.180	0.927
	ND 6h	−0.300	0.113	−2.654	0.207
HFD 1h	HFD 48h	0.767	0.113	6.782	< .001
	HFD 6h	−1.500	0.113	−13.270	< .001
	ND 0h	1.033	0.113	9.141	< .001
	ND 1h	0.767	0.113	6.782	< .001 ***
	ND 48h	0.933	0.113	8.257	< .001
	ND 6h	0.500	0.113	4.423	0.008
HFD 48h	HFD 6h	−2.267	0.113	−20.052	< .001
	ND 0h	0.267	0.113	2.359	0.323
	ND 1h	−3.331e −16	0.113	−2.946e −15	1.000
	ND 48h	0.167	0.113	1.474	0.810
	ND 6h	−0.267	0.113	−2.359	0.322
HFD 6h	ND 0h	2.533	0.113	22.411	< .001
	ND 1h	2.267	0.113	20.052	< .001
	ND 48h	2.433	0.113	21.527	< .001
	ND 6h	2.000	0.113	17.693	< .001 ***
ND 0h	ND 1h	−0.267	0.113	−2.359	0.322
	ND 48h	−0.100	0.113	−0.885	0.983
	ND 6h	−0.533	0.113	−4.718	0.004 ##
ND 1h	ND 48h	0.167	0.113	1.474	0.810
	ND 6h	−0.267	0.113	−2.359	0.322
ND 48h	ND 6h	−0.433	0.113	−3.833	0.025

ANOVA Figure 4 - sWAT

ANOVA - H2AX

Cases	Sum of Squares	df	Mean Square	F	p
V1	80.385	7	11.484	357.9	< .001
Residual	0.513	16	0.032		

Note. Type III Sum of Squares

Post Hoc Tests ▼

Post Hoc Comparisons - V1 ▼

		Mean Difference	SE	t	p_{tukey}
HFD 0h	HFD 1h	−0.067	0.146	−0.456	1.000
	HFD 48h	−3.700	0.146	−25.299	< .001 ###
	HFD 6h	−3.667	0.146	−25.071	< .001 ###
	ND 0h	0.233	0.146	1.595	0.746
	ND 1h	0.233	0.146	1.595	0.747
	ND 48h	−3.467	0.146	−23.704	< .001
	ND 6h	−3.367	0.146	−23.020	< .001
HFD 1h	HFD 48h	−3.633	0.146	−24.843	< .001
	HFD 6h	−3.600	0.146	−24.615	< .001
	ND 0h	0.300	0.146	2.051	0.481
	ND 1h	0.300	0.146	2.051	0.481
	ND 48h	−3.400	0.146	−23.248	< .001
	ND 6h	−3.300	0.146	−22.564	< .001
HFD 48h	HFD 6h	0.033	0.146	0.228	1.000
	ND 0h	3.933	0.146	26.895	< .001
	ND 1h	3.933	0.146	26.895	< .001
	ND 48h	0.233	0.146	1.595	0.746
	ND 6h	0.333	0.146	2.279	0.360
HFD 6h	ND 0h	3.900	0.146	26.667	< .001
	ND 1h	3.900	0.146	26.667	< .001
	ND 48h	0.200	0.146	1.368	0.859
	ND 6h	0.300	0.146	2.051	0.482
ND 0h	ND 1h	2.442e −15	0.146	1.670e −14	1.000
	ND 48h	−3.700	0.146	−25.299	< .001 ###
	ND 6h	−3.600	0.146	−24.615	< .001 ###
ND 1h	ND 48h	−3.700	0.146	−25.299	< .001
	ND 6h	−3.600	0.146	−24.615	< .001
ND 48h	ND 6h	0.100	0.146	0.684	0.996

ANOVA Figure 4 -vWAT

ANOVA - H2AX

Cases	Sum of Squares	df	Mean Square	F	p
V1	228.040	7	32.577	309.0	< .001
Residual	1.687	16	0.105		

Note. Type III Sum of Squares

Post Hoc Tests

Post Hoc Comparisons - V1

		Mean Difference	SE	t	p_{tukey}
HFD 0h	HFD 1h	−0.200	0.265	−0.754	0.993
	HFD 48h	−3.467	0.265	−13.077	< .001###
	HFD 6h	−9.367	0.265	−35.333	< .001###
	ND 0h	0.033	0.265	0.126	1.000
	ND 1h	−0.033	0.265	−0.126	1.000
	ND 48h	−1.533	0.265	−5.784	< .001
	ND 6h	−4.267	0.265	−16.095	< .001
HFD 1h	HFD 48h	−3.267	0.265	−12.322	< .001
	HFD 6h	−9.167	0.265	−34.578	< .001
	ND 0h	0.233	0.265	0.880	0.984
	ND 1h	0.167	0.265	0.629	0.998
	ND 48h	−1.333	0.265	−5.030	0.002
	ND 6h	−4.067	0.265	−15.340	< .001
HFD 48h	HFD 6h	−5.900	0.265	−22.256	< .001
	ND 0h	3.500	0.265	13.203	< .001
	ND 1h	3.433	0.265	12.951	< .001
	ND 48h	1.933	0.265	7.293	< .001***
	ND 6h	−0.800	0.265	−3.018	0.112
HFD 6h	ND 0h	9.400	0.265	35.458	< .001
	ND 1h	9.333	0.265	35.207	< .001
	ND 48h	7.833	0.265	29.549	< .001
	ND 6h	5.100	0.265	19.238	< .001***
ND 0h	ND 1h	−0.067	0.265	−0.251	1.000
	ND 48h	−1.567	0.265	−5.910	< .001###
	ND 6h	−4.300	0.265	−16.220	< .001###
ND 1h	ND 48h	−1.500	0.265	−5.658	< .001
	ND 6h	−4.233	0.265	−15.969	< .001
ND 48h	ND 6h	−2.733	0.265	−10.311	< .001
